# A Disappointment

**Published:** 1888-09

**Authors:** 


### A DISAPPOINTMENT.

Dr. W. C. Barrett is in receipt of a cablegram from Dr. W. D. Miller, in which he states that he has been unavoidably detained at home, and shall have to forego the pleasure of his intended visit to America this fall. We were very much grieved to receive the intelligence, because we had intended to tender Dr. M. a warm reception upon the part of the International Dental Journal Association, which we take the liberty of saying feels honored in counting him one of its members and pledged contributors. There can be no question but that Dr. Miller's contributions in the past in the INDEPENDENT PRACTITIONER have served, more than anything else, to place the journal upon the high plane it now occupies. The series of articles that have just been completed under our management have been of a very high character and reflect credit upon Dr. Miller. They show how vast the field is, and how badly workers are needed to help solve the many yet unknown problems regarding oral diseases. Pyorrhœa Alveolaris still remains a *terra incognita*, having baffled, as yet, all attempts at the determination of its etiology. Still the work progresses, and step by step the sum of our knowledge is augmented. Nothing is impossible to the patient toiler, and we know no field of labor where patient, steady work is more needed than in that of Mycology—never has advance in any science been more laborious than that made in the germ theory of disease.

